# Micromorphological features of brown rotted wood revealed by broad argon ion beam milling

**DOI:** 10.1038/s41598-024-83578-y

**Published:** 2024-12-30

**Authors:** Rikako Tsukida, Tomohiro Hatano, Yuka Kojima, Satoshi Nakaba, Yoshiki Horikawa, Ryo Funada, Barry Goodell, Makoto Yoshida

**Affiliations:** 1 United Graduate School of Agricultural Science, Tokyo University of Agriculture and Technology, Saiwai-cho, Fuchu, Tokyo, 183-8509 Japan; 2 Faculty of Agriculture, Tokyo University of Agriculture and Technology, Saiwai-cho, Fuchu, Tokyo, 183-8509 Japan; 3 JEOL Ltd., Musashino, Tokyo, 196-8558 Akishima Japan; 4 Institute of Agriculture, Tokyo University of Agriculture and Technology, Saiwai-cho, Fuchu, Tokyo, 183- 8509 Japan; 5 Department of Microbiology, University of Massachusetts, Amherst, MA 01003 USA; 6 Sustainable Materials and Technology, SFR, University of Maine, Orono, ME 04469 USA

**Keywords:** Wood degradation, Brown rot fungi, Broad ion beam milling, Ray parenchyma cells, Tracheids, Fungal biology, Fungal ecology, Fungal physiology

## Abstract

Brown rot fungi, the major decomposers in the boreal coniferous forests, cause a unique wood decay pattern but many aspects of brown rot decay mechanisms remain unclear. In this study, decayed wood samples were prepared by cultivation of the brown rot fungi *Gloeophyllum trabeum* and *Coniophora puteana* on Japanese coniferous wood of *Cryptomeria japonica*, and the cutting planes were prepared using broad ion beam (BIB) milling, which enables observation of intact wood, in addition to traditional microtome sections. Samples were observed using field-emission SEM revealing that areas inside the end walls of ray parenchyma cells were the first to be degraded. Osmium reaction precipitates were observed in the degraded regions, as well as in plasmodesmata. In the cell wall where ray parenchyma cells contacted with the tracheids, specific degradation of cross-field pits and hyphal elongation into this area was observed in degradation by both fungi. Other pit types were also degraded as noted in previous studies. Delamination between the S_1_ and S_2_ layers of tracheids, and cracks in the tracheid cell walls were observed. These findings provide new insights into the cell wall degradation mechanisms during the incipient stages of brown rot decay.

**Supplementary Information:**

The online version contains supplementary material available at 10.1038/s41598-024-83578-y.

## Introduction

Brown rot fungi are the most ubiquitous type of wood decay fungi and they are known as the primary wood decomposer in the boreal coniferous forest. Brown rot fungi degrade plant cell wall polysaccharides including cellulose and hemicellulose, while modifying lignin, which is a recalcitrant aromatic polymer^1–3^. Known characteristics of brown rot decay in incipient stages include the preferential degradation of hemicellulose^4^ and the rapid depolymerization of cellulose^5,6^which are both associated with a rapid loss of mechanical properties in brown rotted wood^7,8^.

Morphological studies using optical microscopes^6,9–11^, polarizing microscopes^10,12,13^, and electron microscopes^9,12,14–19^ have revealed that brown rot decay typically begins in the internal layers of the wood cell wall (S_2_ layer), while the cell wall adjacent to the hyphae (S_3_layer) remains intact. In general, the polymer network of cellulose, hemicellulose and lignin in wood cell walls does not have pore sizes sufficient for enzymes to pass through. Based on these facts, it was initially suggested over 60 years ago that degradation by very small enzymes was involved in the degradation mechanisms of brown rot decay^6,20^. It is now recognized that enzymes of this size do not exist, and rather low molecular weight fungal metabolites are employed by brown rot fungi to open the wood cell wall structure^21^. The currently accepted non-enzymatic degradation system is the chelator-mediated Fenton reaction (CMF reaction), in which iron is reduced remotely within the wood cell wall via action of the fungus, and hydroxyl radicals are generated within the wood cell wall from a reaction between hydrogen peroxide and ferrous iron, to oxidatively depolymerize wood cell wall components^21–24^. The CMF reaction proposes that oxalic acid produced by fungi is involved in iron chelation in the fungal extracellular matrix. Low molecular weight aromatic compounds then accept that iron from oxalate, and reduce the ferric iron to ferrous ions once the aromatic compounds have diffused within the wood cell wall (a reaction proposed to be mediated both by the higher pH of the wood cell wall, as well as a reduced concentration of oxalate within the wood cell wall)^21^. Although several genes that could potentially encode enzymes involved in CMF system have been shown to be upregulated in early stages of decay by brown rot fungi^25,26^, it remains unclear which, if any, of the genes shown to be upregulated in that work are actually involved in CMF chemistry, as the derivation of the secondary metabolites responsible for the oxidative action can be challenging to parse out. Further, the CMF mechanism provides a means for protection of the fungus from oxygen radical attack, and issue which has not been adequately addressed in literature focused on only gene expression in brown rot decay^26,27^ and only limited protection of select extracellular enzymes to hydroxyl radical attack has been shown.

Morphological observation of wood decayed by brown rot fungi can be challenging due to its brittleness, which can lead to the loss of the information during sample preparation. Transmission electron microscopy (TEM) has been used to observe decayed wood which has been fixed and embedded with resins^15–17^, which allows observation with high-resolution, but provides only a limited range of observation due to the grid. In contrast, scanning electron microscopy (SEM) allows continuous observation with a wide range of magnification, and recently, its resolution has advanced to a level comparable to that of the TEM^28^. We have recently established a method that combines field-emission SEM (FE-SEM) with broad ion beam (BIB) milling based on sputtering^29^, which enables observation of intact wood, and this method can be applied to the observation of decayed wood, which has lost its strength and become brittle.

The present study focuses on the decay phenomena in softwoods, which many species of brown rot fungi preferentially attack. Morphological changes during the decay process in the tracheids, which make up the largest proportion of softwood tissue, were observed, as well as morphological changes in the ray parenchyma where decay fungi are known to attack initially during incipient stages of wood colonization^9,14,30,31^. We used Japanese cedar (*Cryptomeria japonica*), a representative conifer with high economical value in Japan, as the substrate and decayed it with the brown rot fungi *Gloeophyllum trabeum* and *Coniophora puteana*. In addition to a conventional section preparation using an ultra-microtome, we employed BIB-milled cross-sectioning and observed the samples using FE-SEM. Through this approach, we reevaluated the degradation process of wood cell walls in the early stage of brown rot decay.

## Results

### Low-magnification observation of the decayed wood samples

**Fig. 1:**
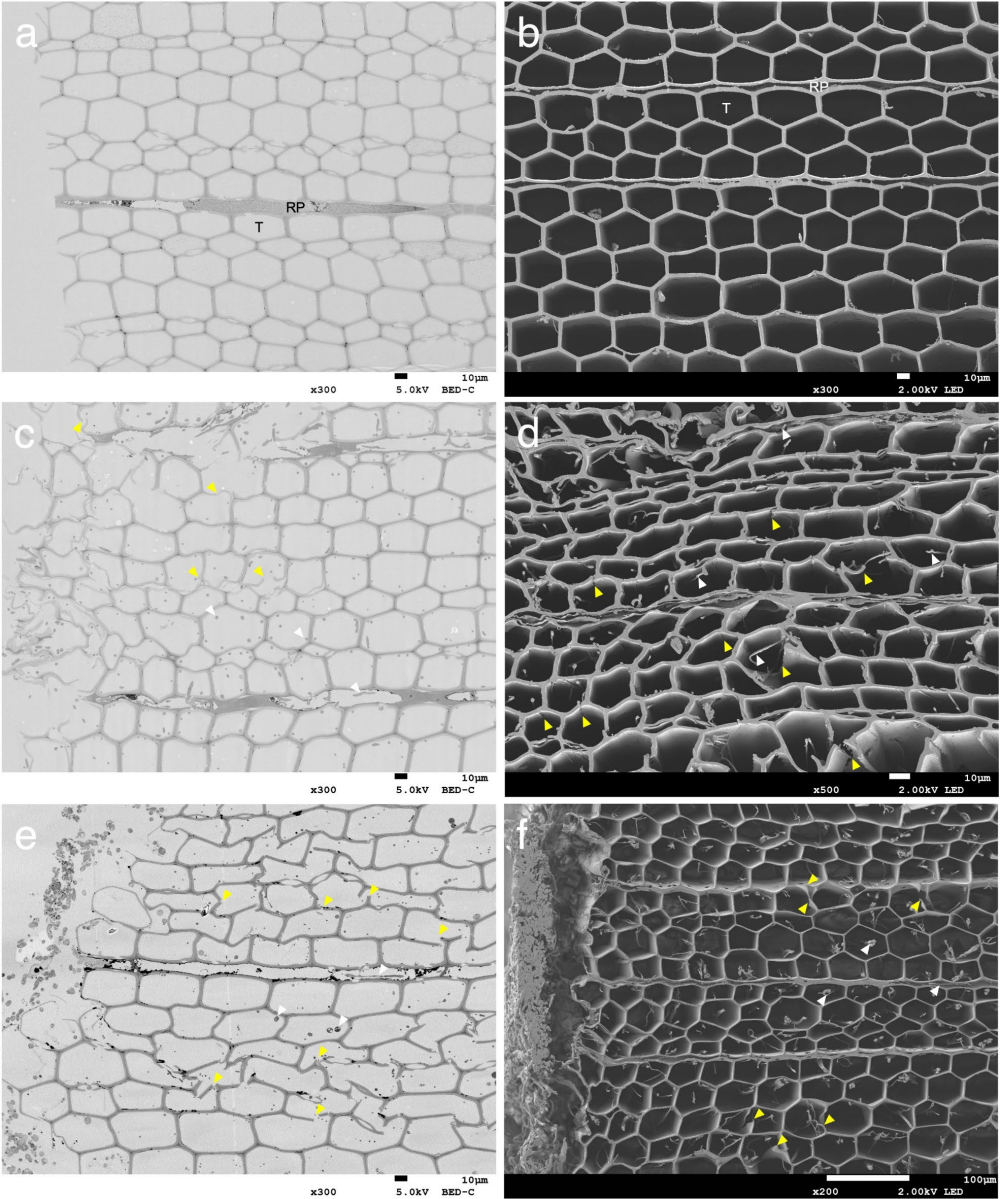
Low-magnification transverse section images: (**a**,**b**) undecayed wood, (**c**,**d**) *G. trabeum* 32 days, (**e**,**f**) *C. puteana* 12 days. Images a, c and e are sections prepared using an ultra-microtome. Images b, d and f are cross-sections obtained through BIB milling. White arrowheads indicate hyphae. Yellow arrowheads indicate cracks. RP = ray parenchyma; T = tracheid.

The mass loss rates of the undecayed (control) and decayed wood were estimated using additional samples that had been decayed under the same conditions with those for microscopic observation (Supplementary Fig. 1). Transverse sections of wood samples were observed using FE-SEM at low magnification. In the undecayed wood, intact tracheids and traversing ray parenchyma were observed (Fig. 1a,b). When samples decayed by *G. trabeum* for 32 days and by *C. puteana* for 12 days were observed, hyphal colonization of the ray parenchyma and tracheid lumens was noted, with the hyphae extending from more heavily colonized regions into uninfected wood tissue (Fig. 1c–f). Observation of the ultra-microtome sections using a diamond knife revealed that while some wood cells appeared normal, distortions in the honeycomb-like structure of the tracheids and cracks in the wood cell walls were observed because of the stresses produced when the microtome knife cut through the samples (Fig. 1c,e). Particularly, mechanical stress during microtome cutting of decayed wood cell walls appeared to promote changes in the shape of the cells and this would therefore have also distorted some micro- and nano-scale features of the cell walls. The BIB milling method, which does not induce mechanical stress was therefore tested in comparison to ultra-microtome sections, a conventional method. The BIB-milled cross-sections appeared to improve structural integrity with less damage to the surface particularly in decayed sample material, and in samples where both decayed and undecayed areas were present (Fig. 1d,f). Interestingly, when observing wood samples decayed by *C. puteana* using the BIB milling method, although many cracks were observed (Fig. 1f and Supplementary Fig. 2), the distortion of the cellular structure was relatively mild compared with microtome sections (Fig. 1e). Considering the mechanical stresses applied in the microtome sections, the BIB milling method appears to provide sample surfacing with less distortion of the sample, particularly when transitions from dense to lower density material occurs, or when the material is already at low density, in our case because of decay.

### Degradation of ray parenchyma cells

**Fig. 2:**
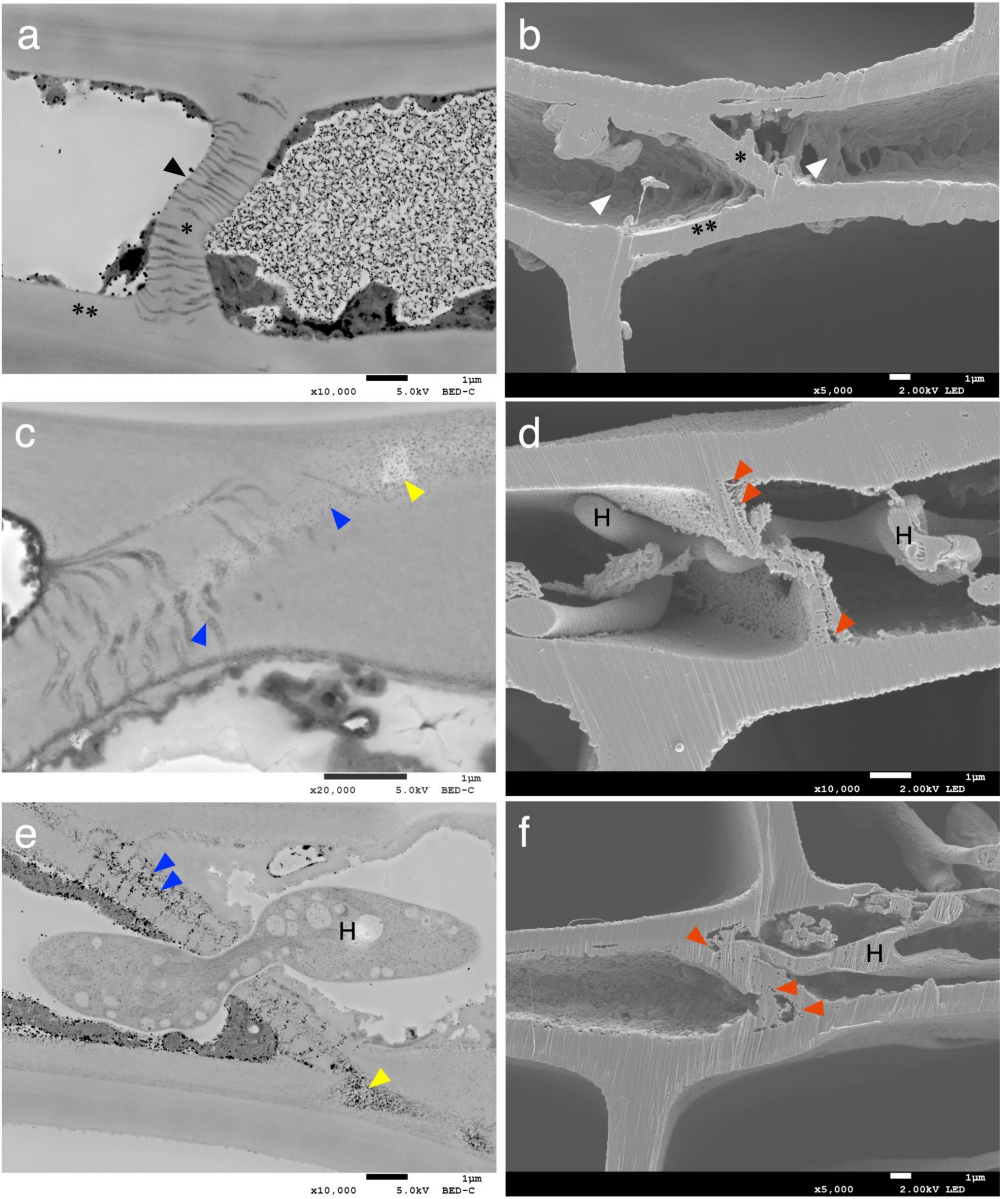
Observation of ray parenchyma cells: (**a**,**b**) undecayed wood, (**c**,**d**) *G. trabeum* 32 days, (**e**) *C. puteana* 32 days, (**f**) *C. puteana* 12 days. Images a, c and e are sections prepared using an ultra-microtome. Images b, d and f are cross-sections obtained through BIB milling. A black arrowhead indicates plasmodesmata. White arrowheads indicate cellular content in the ray parenchyma cells. Blue arrowheads indicate osmium-reaction precipitates (ORPs). Yellow arrowheads indicate the areas appeared to have greater transparency. Red arrowheads indicate degradation areas within the end wall, including areas where small pores, and coalesced pores were created as part of the decay process. H = hyphae; * = end walls; ** = the cell walls of ray parenchyma cells in contact with tracheids.

Unlike tracheids, ray parenchyma cells remain alive for a long time after differentiation^32,33^, and the cell walls of ray parenchyma in *C. japonica *possess only a primary cell wall^34^. Observations of ultra-microtome sections showed that cell contents were present in some cell lumens that stained with osmium tetroxide, but in other cases the cell lumens were empty and did not stain (Fig. 2a). Observations of BIB-milled cross-sections showed cellular content adhering to the surface of the cell lumen (Fig. 2b) which potentially could be due to the effect of the critical point drying treatment used in the BIB milling method. In decayed wood samples, as revealed by BIB-milled cross-sections, when hyphae were present colonizing a ray parenchyma cell, that cell contained no cellular content, suggesting that the fungus metabolized the residues stored in these parenchyma cells (Fig. 2d,f).

**Fig. 3:**
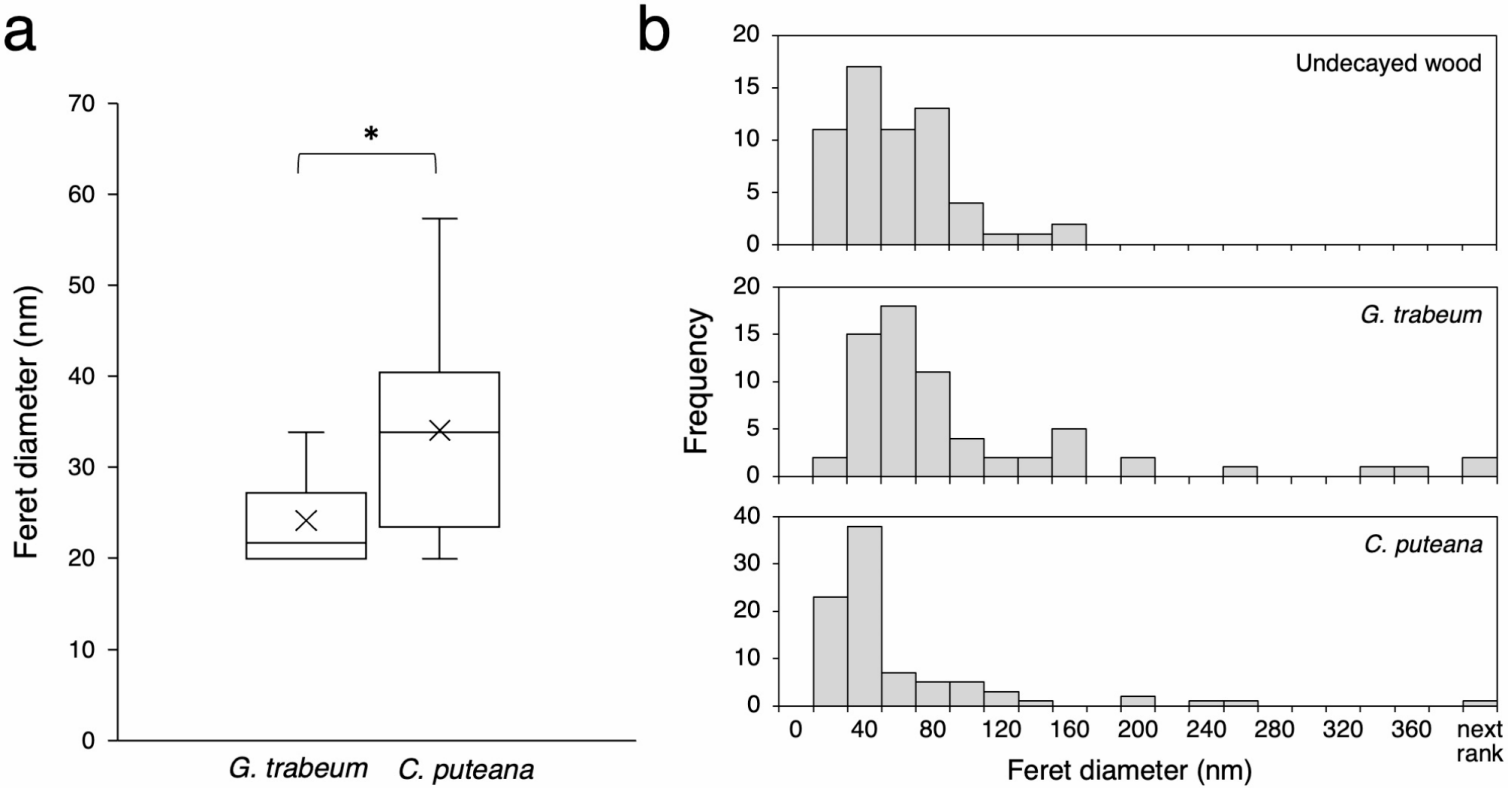
Image analysis of the end walls of ray parenchyma cells. (**a**) The size of ORPs in the decayed wood samples by *G. trabeu*m 32 days and *C. puteana* 32 days. Statistical significance was calculated using a Welch’s t-test. *P < 0.05. (**b**) The size of the pores in the end walls of ray parenchyma cells in Fig. 2b,d,f, respectively. Pores larger than 400 nm were included in the next rank.

Individual ray parenchyma cells are separated from adjacent ray parenchyma cells by their end walls, and also from tracheids by the cell walls (Fig. 2a,b). The end wall is known to possess micropores with a diameters of several tens of nm, called plasmodesmata, which are absent in walls that contact the tracheids^35,36^. The plasmodesmata of the ray parenchyma cells stained dark when microtome sections were observed (Fig. 2a). Observation of decayed wood sections revealed a number of osmium-reaction precipitates (ORPs) in the plasmodesmata, and the precipitate particles extended into the middle lamella (Fig. 2c,e). Similar particles have also been observed in previous studies of decayed wood stained with osmium^37–39^. Although it is unknown what compounds were stained by osmium, measurements of precipitate size in decayed wood samples by *G. trabeum* and *C. puteana* showed that the average Feret diameter was 24 nm and 34 nm, respectively (Fig. 3a and Supplementary Fig. 3), suggesting that the precipitate size varies by fungal species in the same wood. However, it should be noted that the possibility cannot be ruled out that the *C. puteana* decayed samples more aggressive decay compared to the *G. trabeum* decayed samples (Supplementary Fig. 1), and this may be reflected in the ORP size. In the middle lamella, the regions with ORPs displayed reduced electron density and these areas appeared to have greater transparency when imaging (Fig. 2c,e). When observed BIB-milled cross-sections, small pores were observed in the corresponding reduced electron density regions observed in microtome sections (Fig. 2d,f). Although pores were sparsely present in the end walls of the undecayed wood (27 ~ 216 nm), larger sized pores were present due to decay (27 ~ 675 nm) (Fig. 3b and Supplementary Fig. 4), indicating that the porosity of the end walls increases during the decay process. In contrast, the cell walls of ray parenchyma cells contacting with tracheids in the same samples were not degraded and had no change in porosity. These findings suggest that hyphae invade the ray parenchyma first, attack the end walls, and then ramify into neighboring ray parenchyma cells.

### Hyphal elongation between ray parenchyma and tracheids

**Fig. 4:**
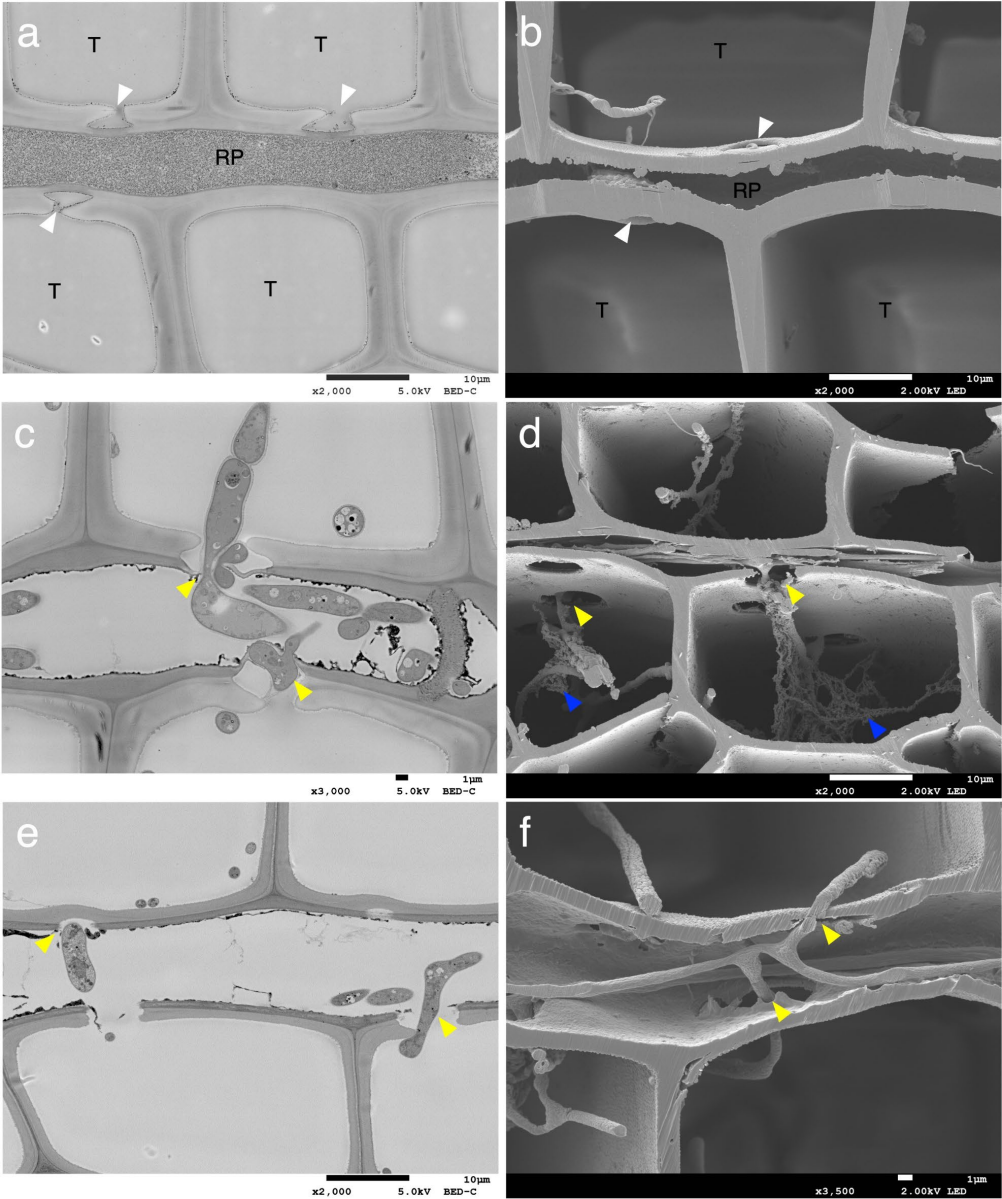
Observation of the cross-field pitting region. (**a**,**b**) undecayed wood. (**c**,**d**) *G. trabeum* 32 days. (**e**,**f**) *C. puteana* 12 days. Images a, c and e are sections prepared using an ultra-microtome. Images b, d and f are cross-sections obtained through BIB milling. White arrowheads indicate cross-field pits. Yellow arrowheads indicate hyphal elongation through cross-field pits. Blue arrowheads indicate characteristic materials around hyphae. RP = ray parenchyma; T = tracheid.

Cross-field pitting between ray parenchyma cells and tracheids^40^ was observed in both undecayed (Fig. 4a,b) and decayed samples (Fig. 4c–f). In the decayed wood samples, under conditions where the cell walls of ray parenchyma cells and tracheids were relatively intact, hyphal elongation through cross-field pits was observed to be associated with degradation of the surrounding wood (Fig. 4c–f). Decay fungi are known to preferentially pass through pits or bore holes in the cell walls during initial stages of wood colonization as the hyphae elongate and ramify into neighboring wood cells^6,13,41,42^. In addition to hyphal passage through bordered pits and simple pits therefore, we suggest that cross-field half-bordered pits are also initially used in hyphal elongation between ray parenchyma and tracheids.

The BIB milling method revealed that the fungal cell wall of *G. trabeum* consisted of a densely packed inner layer and a relatively loose outer layer known as the extracellular matrix (ECM) (Fig. 4d)^43^. The ECM has been observed in mature hyphae, hyphal tips and branching points, but not in senescing or dead hyphae^44,45^, suggesting therefore that the fungi observed were alive and active. Similar features were also observed in the ultra-microtome sections, where a matrix-like material within the lumen of the cell walls was observed around the hyphae, and that also adhered to the inner layer of the wood cell walls (Supplementary Fig. 5a). The ECM is believed to play a role in substrate recognition, adhesion and creating an environment suitable for enzyme and metabolite reactions^43^, and controlling how, or whether, metabolites can diffuse through the ECM to the wood cell wall^46^. A similar ECM was also observed in *C. puteana* in a thin layer on the surface of the hyphae (Supplementary Fig. 5b), suggesting that the adhesion of the ECM to the wood cell walls is part of the active degradation process in brown rot decay.

### Delamination between S_1_ and S_2_ layers in the tracheid cell wall

**Fig. 5:**
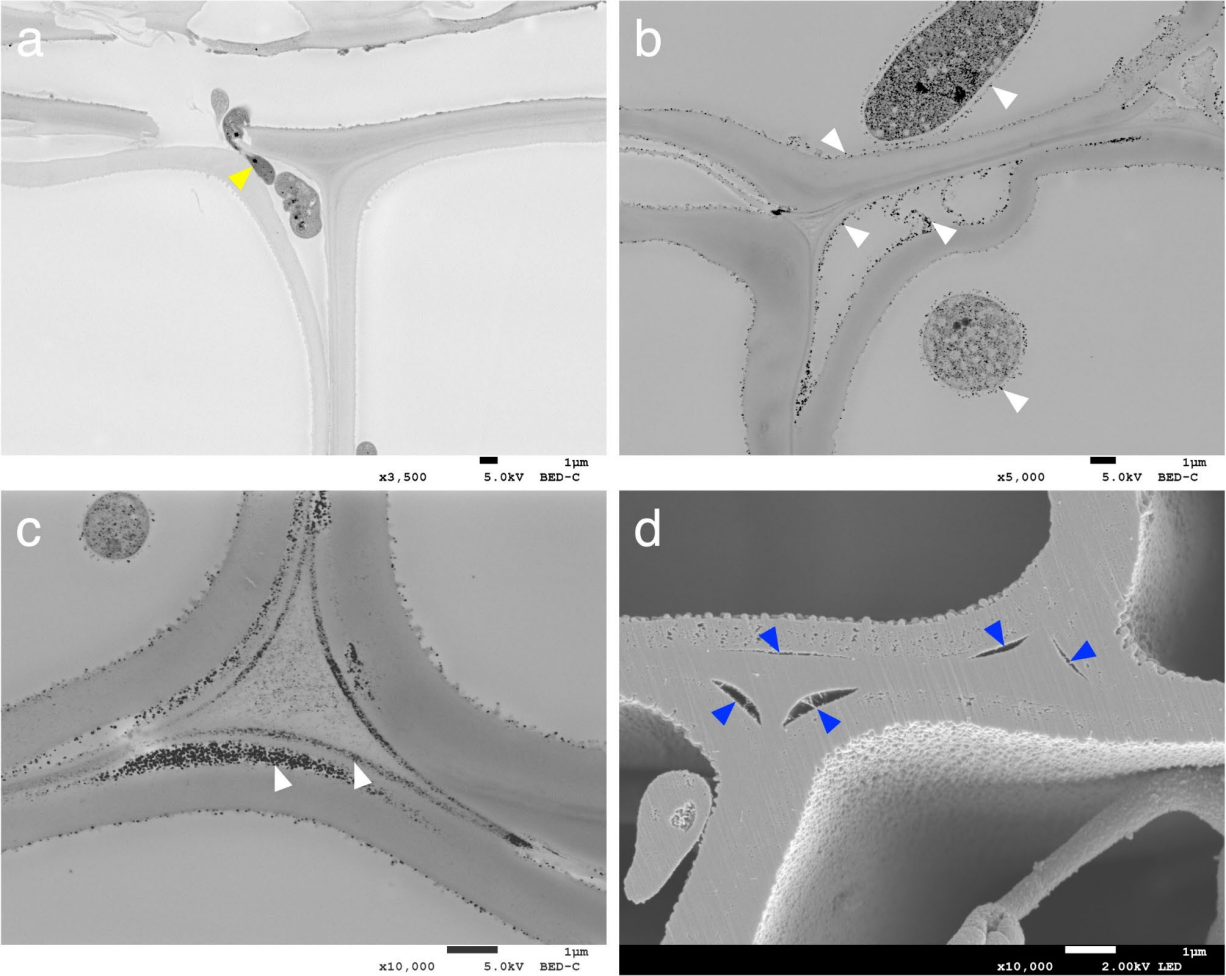
Delamination between the S_1_ and S_2_ layers in tracheid cell walls: (**a**) *G. trabeum* 12 days, (**b**,**c**) *C. puteana* 16 days, (**d**) *G. trabeum* 32 days. Images a–c are sections prepared using an ultra-microtome. Image d shows cross-sections obtained through BIB milling. A yellow arrowhead indicates hyphae elongation into the delamination area. White arrowheads indicate ORPs. Blue arrowheads indicate delamination.

In the tracheids of wood decayed by both fungal species, delamination was often observed occurring between the S_1_ and S_2_ layers (Fig. 5a,b). Similar delamination has been noted in a previous study^47^, and there have also been reports that degradation of the wood cell wall initiated in this region^16^. However, although delamination could have been caused during section preparation using an ultra-microtome, similar delamination was also observed when using the BIB milling method (Fig. 5d). This strongly suggests that the delamination was induced by fungal degradation. Compared to the BIB-milled cross-sections, however, delamination in sections using the ultra-microtome was more pronounced, suggesting that the mechanical stress caused by the microtome’s diamond knife further promoted delamination. In the wood samples decayed by *G. trabeum*, hyphal elongation into the delamination void was observed (Fig. 5a). This occurred in tracheids adjacent to ray parenchyma, suggesting that the hyphae grew and extended from the ray parenchyma into the delamination zone. Hyphal elongation between the S_1_ and S_2_ layer may therefore also be one of the main pathways for invasion from ray parenchyma into the tracheids before the significant degradation of the remainder of the wood cell wall.

In *C. puteana* decayed wood, ORPs were observed in the delamination zone between the S_1_ and S_2_ layers (Fig. 5b). The precipitates were observed between the S_1_ and S_2_ layers even in the absence of notable delamination (Fig. 5c), and on the surface of the outer S_1_ layer (Fig. 5b,c). Although it is unclear from this study what the osmium stained and why precipitates formed, the fact that ORPs were observed in layers only in the decayed wood samples suggests that structural or compositional changes occurred in this region during the decay process.

## Discussion

It is known that brown rot fungi have lost or greatly reduced the genes encoding lignin-degrading enzymes and cellobiohydrolases, the latter being key enzymes in crystalline cellulose degradation, during their evolution^48,49^. As the enzymatic degradation system in brown rot fungi was reduced a more efficient non-enzymatic degradation system known as the CMF reaction evolved^20,22–24^. The CMF reaction employs low-molecular-weight compounds that are small enough to diffuse through the pores of the intact matrix of wood cell walls. Thus, the CMF reaction has been accepted to explain the characteristic decay patterns produced by brown rot fungi, where the S_2_ layer undergoes degradation while the S_3_ layer near the hyphae remains intact. In our current experimental work, although preferential decomposition of the S_2_ layer was not observed in the tracheid cell walls with either fungus, we did observe a preferential delamination at the interface of the S_1_ and S_2_ layers (Fig. 5a,b,d). This suggests that the samples observed in this study, particularly those up to 16 days for *C. puteana* and 32 days for *G. trabeum*, were in an early S_2_ layer decomposition stage. Brown rot fungi have been characterized as causing mechanical property loss in the incipient stages of wood decay because significant mechanical property losses have been observed with weight loss less than 10%^7,8^. In this study, cracks and delamination between the S_1_ and S_2_ layers in the tracheid cell walls were observed in both decayed wood samples not only in the microtome sections (Figs. 1c,e and 5a,b) but also with the BIB milling method (Figs. 1d,f and 5d and Supplementary Fig. 2), which does not apply mechanical stress. These cracks and delamination therefore appear to occur during incipient stages of decay, because of the degradation of cell wall components at that interface before significant degradation of the wood cell walls is observed at the microscopic level. Such damage to the wood cell walls would likely contribute to mechanical property loss in the wood cell wall caused by the brown rot fungus. In the zone between the S_1_ and S_2_ layers, delamination was occasionally associated with hyphal elongation into the delamination zone (Fig. 5a), suggesting that this region had undergone sufficient deterioration to allow fungal invasion. The transition layer (S_12_ layer) between the S_1_ and S_2_layers is an area where the orientation of cellulose microfibrils gradually shifts^50^ and it has high hemicellulose content^51,52^. Since brown rot fungi are known to degrade hemicellulose significantly prior to cellulose in the incipient stages of decay^1–3,53^, the degradation of hemicellulose may be related to the occurrence of delamination. Brown rot CMF chemistry has been demonstrated to preferentially attack amorphous cellulose/hemicellulose regions prior to crystalline cellulose^47,54^. However, since hemicellulases are too large to penetrate the intact S_3_ layer it will be necessary in future research to determine how, or if, hemicellulases penetrate into the wood cell wall in association with, or after the CMF reaction on the wood cell wall.

In the current study, consistent with previous reports^9,14,30,31^, hyphal invasion of the ray parenchyma was observed in early decay stages caused by both fungi. Moreover, significant degradation of the end walls of ray parenchyma cells was often observed in early decay stages; however, even within the same cell, the walls in contact with the tracheids remained intact (Fig. 2d,f). This finding further supports the understanding that the end walls of ray parenchyma cells are the first degraded in decay fungal colonization process. Significant degradation was observed within the end walls of ray parenchyma cells including middle lamella cell corners and ORPs were observed. The fact that the ORPs were prominently observed where degradation was ongoing (Fig. 2c,e) suggests that these ORPs are related to degradation and they may be indicators of specific substrate degradation or potentially are unique fungal metabolites. In addition, ORPs were also observed in the plasmodesmata only in wood samples that were undergoing active decay. In living plants, a defense response occurs when a plant is infected by plant pathogens, and it is known that plasmodesmata are closed by callose deposition when plants are subject to microbial attack^55,56^. However, the ray parenchyma cells were no longer alive in our wood samples so callose deposition would not be possible.

Our observation that brown rot fungi will utilize the plasmodesmata micropores of the wood cells to facilitate penetration of the end walls of ray parenchyma cells is a novel finding (Fig. 2c,e). Although we were unable to identify the specific wood or fungal components that reacted with osmium tetroxide to produce the ORPs, this compound is known to react with phenols^57^, it may have reacted with phenolic derivatives secreted by the fungus, or with degradation products such as lignin fragments. Brown rot fungi are known to produce hydroquinone as a secondary metabolite, which has been shown to be directly involved in the CMF reactions^58–61^, and brown-rot degraded lignin surfaces also are thought to participate in, and enhance, the brown rot CMF reaction^62^. The penetration of low-molecular-weight fungal-derived compounds into the plasmodesmata, with associated oxidation of cellular components, could have promoted the degradation of the end walls of ray parenchyma cells to prepare a pathway for the penetration of hyphae followed by further ramification into neighboring ray parenchyma cells^9,14,30,31^. Hyphal elongation through pits^6,13,41,42^ was also observed in our current study, and the degradation of cross-field pits between the ray parenchyma cells and tracheids was found to be associated with hyphal elongation through the pits in many cases (Fig. 4c–f). These results suggest that brown rot fungi preferentially use and degrade pores such as end wall plasmodesmata of ray parenchyma cells and both ray parenchyma cells and tracheid pits to move through wood before causing significant wood cell wall degradation. Degradation of cell wall material was associated with the exploitation of these pores and permitted the fungal hyphae to spread throughout wood tissue.

## Conclusions

In this study, we performed microscopic observations of brown rotted wood samples at an early stage of decay where hyphae moved through ray parenchyma preferentially and elongated from ray parenchyma into tracheids. As a result, we found morphological features such as the degradation of the plasmodesmata in the end walls of ray parenchyma cells, rupturing of tracheid cell walls, and delamination between the S_1_ and S_2_ layer in the tracheid cell wall, to be important steps necessary in advancing the decay process. Brown rot fungi are known to preferentially decompose the S_2_ layer in incipient stages of decay^13,15,17^; however, the results of this study strongly suggest that the features described above are the key morphological changes which occur in wood undergoing brown rot early decay. It is possible however, that the various morphological features observed in this study may be phenomena that occur before preferential decomposition of the S_2_ layer by brown rot fungi. If so, our findings may also be CMF-related phenomena, since the size of extracellular fungal enzymes are too large to diffuse through the intact wood cell wall, suggesting that the wood cell wall was altered by low-molecular weight compounds. In our current research we employed the BIB milling method, which can be used with little or no damage to the wood cell wall compared to conventional section preparation using an ultra-microtome with observation using high-resolution FE-SEM. Our results confirmed many important characteristics of brown rot decay but also revealed new findings on how the wood cell wall is attacked by brown rot fungi in the initial stages of decay. Further research is required to understand the importance of the osmium-reactive precipitates (ORPs), and whether this reaction product is associated with CMF metabolites, or if the reaction is associated with lignin degradation by the brown rot fungi. Understanding why the precipitate size varies with the species of decay fungi, or whether this phenomenon is just associated with the length of decay time, may also provide insights into how brown rot fungi decay wood.

## Methods

### Fungi used in this study

*G. trabeum* NBRC 6430 obtained from the National Institute of Technology and Evaluation (Kazusa, Japan). *C. puteana* MAFF 420262 obtained from the National Agriculture and Food Research Organization (Tsukuba, Japan). *G. trabeum* and *C. puteana* were incubated at 25 °C and 22 °C, respectively, throughout this study.

### Preparation of decayed wood samples

The fungi described above were inoculated on potato dextrose agar medium. After the mycelia covered the entire surface of the medium, a sterilized plastic net was placed over the surface, and blocks of Japanese cedar (*C. japonica*) sapwood (5 mm (T) × 5 mm (R) × 10 mm (L)), autoclaved at 121 °C for 20 min, were then placed on the net with their tangential faces down, and the blocks then incubated for up to 36 days. Sterile wood blocks placed on uninoculated plates were also used as controls. Mass loss rate during the decay period was estimated using additional samples decayed under the same conditions (Supplementary Fig. 1). Note that mass loss with *G. trabeum* was limited in this condition but this is more typical of decay in *C. japonica *which is a moderately decay resistant wood species, and even sapwood has some resistance to decay in laboratory decay tests^42,63,64^. Mass of the samples before and after incubation was measured after drying the samples at 60 °C for 2 days.

### Microscopic observations

In this study, observations were primarily conducted on decayed wood with an assumed weight loss of 5% or less, focusing on samples that had decayed for 32 days for *G. trabeum* and 12 to 16 days for *C. puteana*. BIB-milled cross-sectioning and ultra-microtome sections were used for the preparation of samples, followed by SEM observation^29^.

The decayed wood samples were immersed in 2.5% glutaraldehyde in sodium phosphate buffer (0.1 M) (pH 7.4) overnight at 4 °C. Then the samples were rinsed (5x) with sodium phosphate buffer (0.1 M) (pH 7.4) and subsequently postfixed in 1.0% OsO_4_ in sodium phosphate buffer (0.1 M) (pH 7.4) for 2 h at room temperature. The samples were then rinsed 3 times with sodium phosphate buffer (0.1 M) (pH 7.4). Finally, the samples were dehydrated in a graded series of ethanol from 30 to 100% ethanol for 15 min at each step, before being cross-sectioned or sectioned as described below.

For BIB-milled cross-sectioning, the samples were immersed in 100% ethanol and processed in a critical point dryer (SYSGLCP-8, Sanyu-Gijutsu, Akiruno, Japan) with a purging flow rate of 1.0 L/min at 40 °C. The critical point dried samples were mounted on a copper foil (99.95% purity, 0.1-mm-thick, Nilaco, Tokyo, Japan) with a two-component epoxy resin adhesive (Quick 5, Konishi, Osaka, Japan) and subsequently attached to the BIB milling system (IB-19520, JEOL, Akishima, Japan). BIB milling was performed at an accelerating voltage of 4 kV and milling time of 8 h. The cutting planes of BIB milling were coated with osmium (1.5 nm) using an osmium plasma coater (HPC-20, Shinkuu device, Mito, Japan). The coated samples were observed under a scanning electron microscope (JSM-7900F, JEOL) at an accelerating voltage of 2 kV. Images were taken using a secondary electron detector.

For ultra-microtome sections, the samples were immersed in a graded series Spurr epoxy resin (Polysciences, Warrington, PA, USA), in ethanol (1:1 and 2:1 Spurr resin) under rotation for 2 h each at room temperature before a final immersion in 100% Spurr resin with rotation overnight, and then polymerization at 70 °C for 8 h. The embedded samples were cut with an Ultra-microtome (UltraCut UCT Type 706200, Leica, Germany) to a  200 nm section thickness with a 45° diamond knife (Diatome, Helmstrasse, Switzerland). The sections were put on a silicon wafer and observed under a scanning electron microscope at an accelerating voltage of 5 kV. Images were taken using a backscattered electron detector. Image analysis was conducted using ImageJ software (National Institutes of Health, Bethesda, Maryland, USA)^65,66^. The size and number of the osmium-reaction precipitates or pores were obtained by thresholding and binarizing from the contrast of the backscattered electron images or secondary electron images.

## Electronic Supplementary Material

Below is the link to the electronic supplementary material.

Supplementary Material 1

## Data Availability

All data generated or analyzed during this study are included in this article and supplemental data. The data of this study are available from Makoto Yoshida (the corresponding author) upon request.
