# Supplementary material for: Micromorphological features of brown rotted wood revealed by broad argon ion beam milling
Source: Sci Rep. 2024 Dec 30;14:32003. doi: 10.1038/s41598-024-83578-y (PMC11686018; doi:10.1038/s41598-024-83578-y)
Supplement: Supplementary file 1 — Supplementary Material 1 [file 41598_2024_83578_MOESM1_ESM.pdf]

Supplementary materials for

Micromorphological features of brown rotted wood revealed by broad argon ion beam milling

Rikako Tsukida, Tomohiro Hatano, Yuka Kojima, Satoshi Nakaba, Yoshiki Horikawa, Ryo Funada, Barry Goodell, and Makoto Yoshida

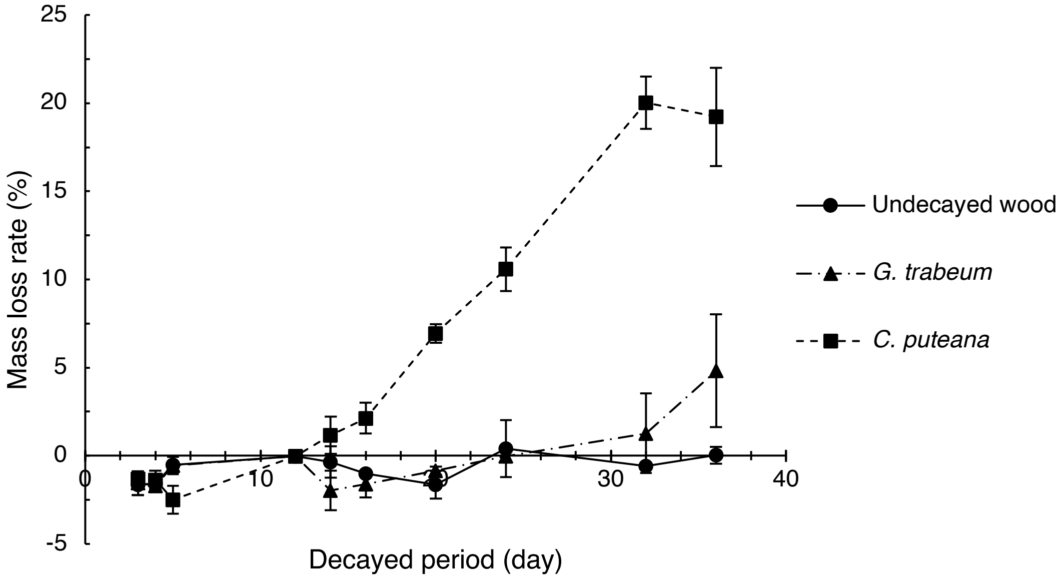

Supplementary Fig. 1. Mass loss rate of the incubated wood samples. The experiments were repeated four times.

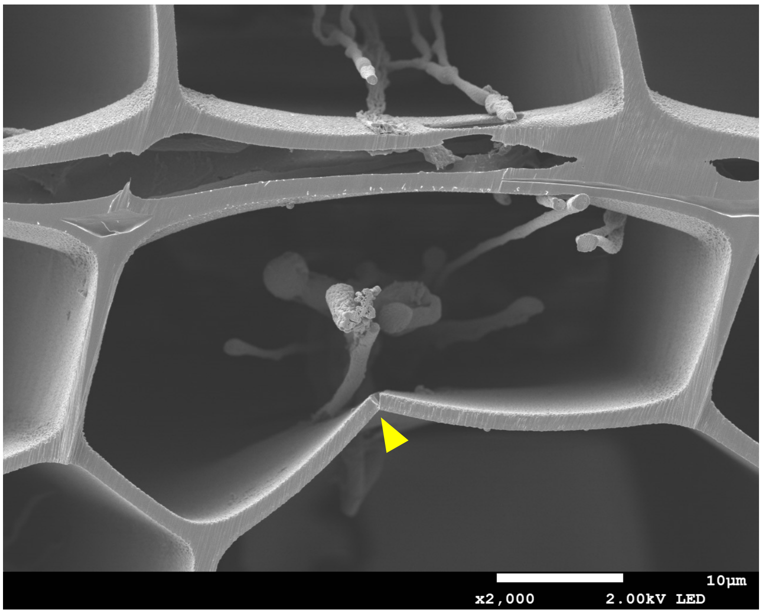

Supplementary Fig. 2. BIB-milled cross-section of wood sample decayed by *C. puteana* for 12 days.

A yellow arrowhead indicates crack.

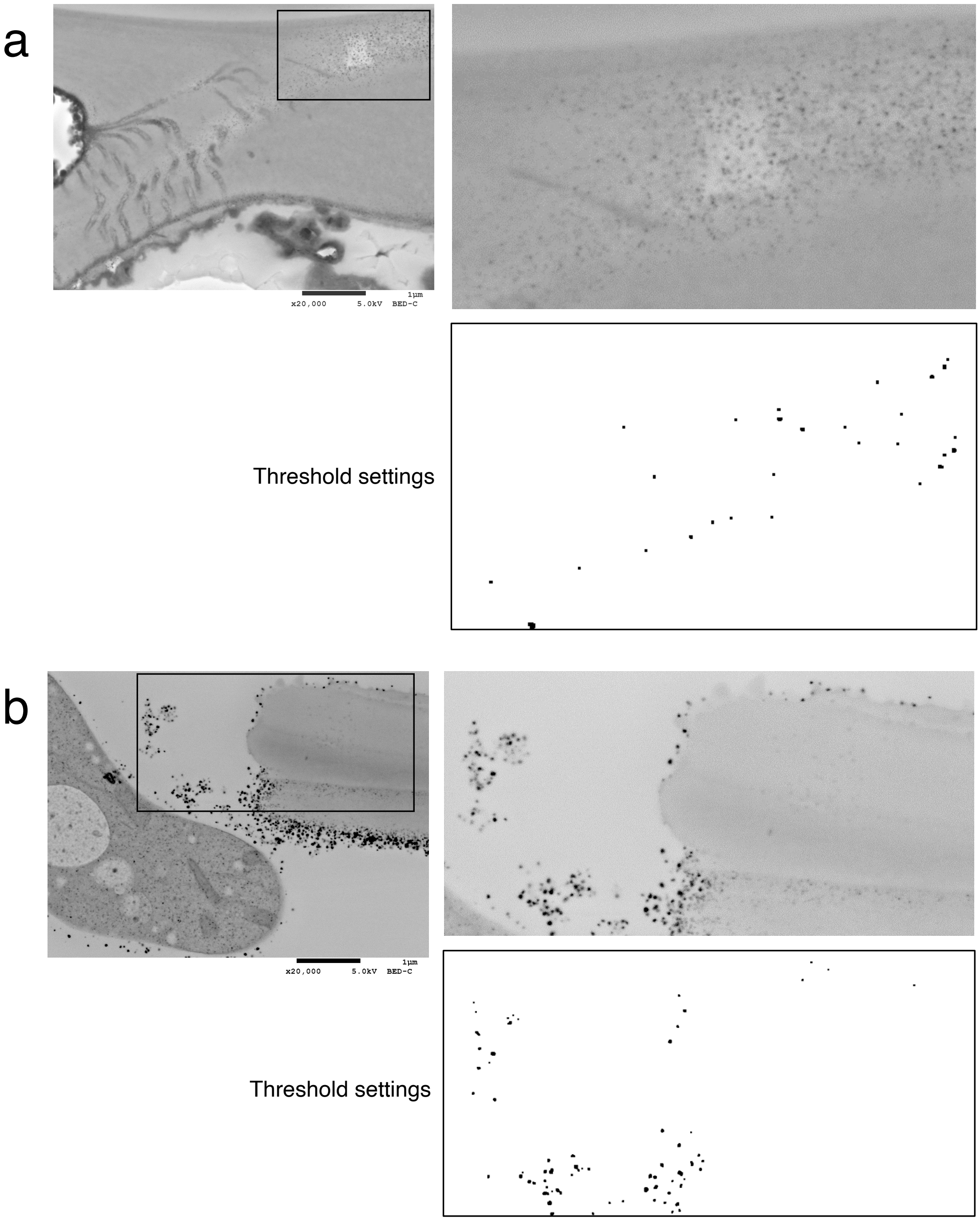

Supplementary Fig. 3. Threshold setting in the measurement of the size of ORPs. a) a sample decayed by *G. trabeum* 32 days, b) a sample decayed by *C. puteana* 32 days.

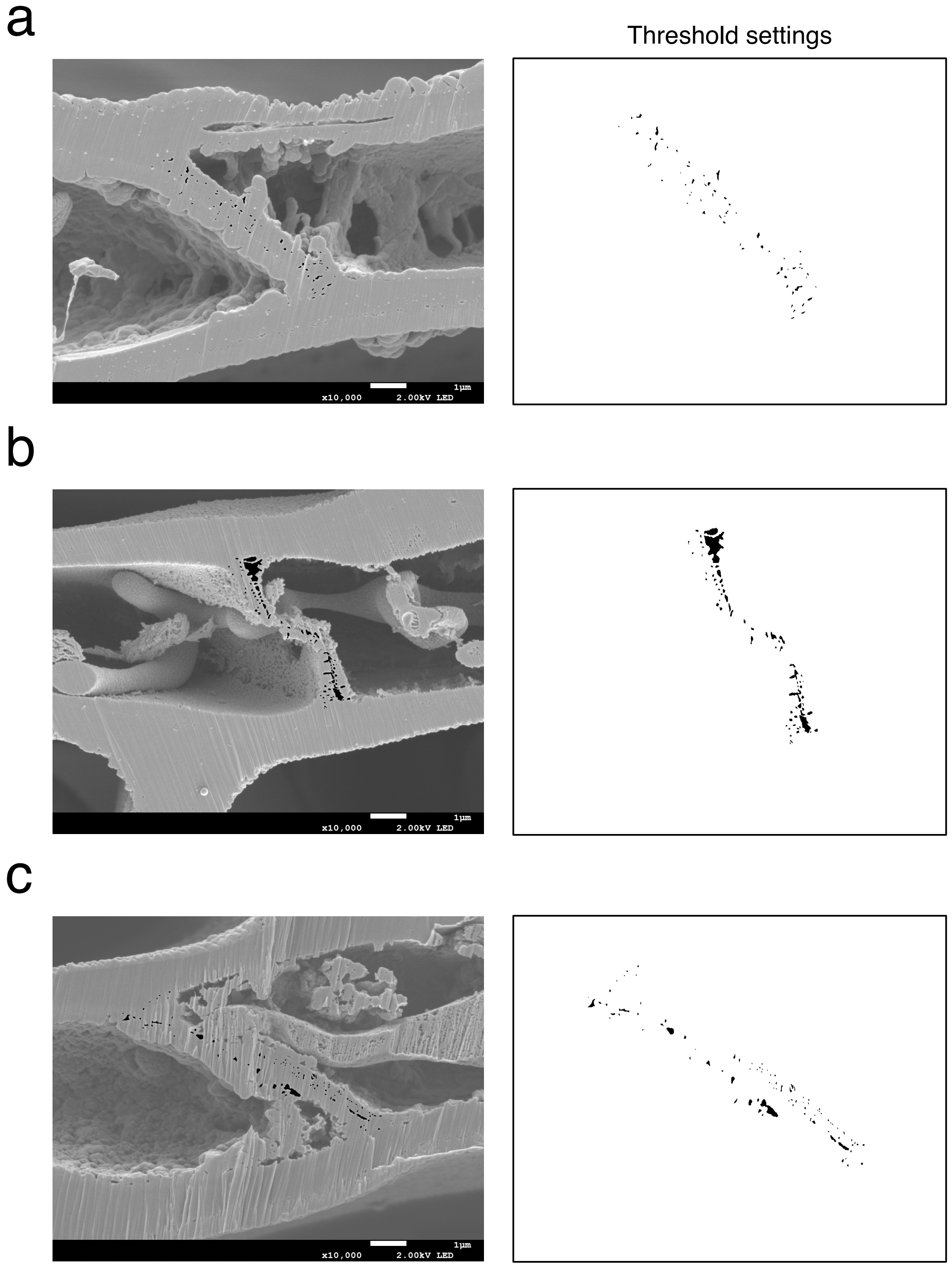

Supplementary Fig. 4. Threshold setting in measurement of the pore size. a) undecayed wood, b) a sample decayed by *G. trabeum* 32 days, c) a sample decayed by *C. puteana* 12 days.

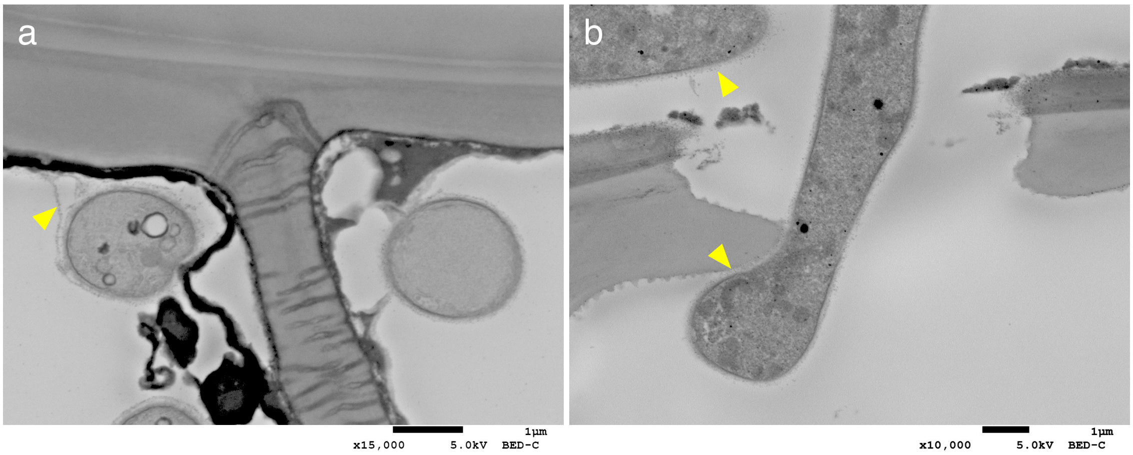

Supplementary Fig. 5. Example of the ECM observed in sections prepared using an ultra-microtome.

a) *G. trabeum* 32 days, b) *C. puteana* 12 days. Yellow arrowheads indicate the ECM.
